# Metabolic engineering of *Synechococcus elongatus* PCC 7942 for improvement of 1,3-propanediol and glycerol production based on in silico simulation of metabolic flux distribution

**DOI:** 10.1186/s12934-017-0824-4

**Published:** 2017-11-25

**Authors:** Yasutaka Hirokawa, Shingo Matsuo, Hiroyuki Hamada, Fumio Matsuda, Taizo Hanai

**Affiliations:** 1 Laboratory for Bioinformatics, Graduate School of Systems Biosciences, Kyushu University, 804 Westwing, 3-1-1 Maidashi, Higashi-ku, Fukuoka, 812-8582 Japan; 2 Department of Bioinformatic Engineering, Graduate School of Information Science and Technology, Osaka University, 1-5 Yamadaoka, Suita, Osaka 565-0871 Japan

**Keywords:** Cyanobacteria, Flux balance analysis, Synthetic metabolic pathway, 1,3-Propanediol

## Abstract

**Background:**

Production directly from carbon dioxide by engineered cyanobacteria is one of the promising technologies for sustainable future. Previously, we have successfully achieved 1,3-propanediol (1,3-PDO) production using *Synechococcus elongatus* PCC 7942 with a synthetic metabolic pathway. The strain into which the synthetic metabolic pathway was introduced produced 3.48 mM (0.265 g/L) 1,3-PDO and 14.3 mM (1.32 g/L) glycerol during 20 days of incubation. In this study, the productivities of 1,3-PDO were improved by gene disruption selected by screening with in silico simulation.

**Methods:**

First, a stoichiometric metabolic model was applied to prediction of cellular metabolic flux distribution in a 1,3-PDO-producing strain of *S. elongatus* PCC 7942. A genome-scale model of *S. elongatus* PCC 7942 constructed by Knoop was modified by the addition of a synthetic metabolic pathway for 1,3-PDO production. Next, the metabolic flux distribution predicted by metabolic flux balance analysis (FBA) was used for in silico simulation of gene disruption. As a result of gene disruption simulation, NADPH dehydrogenase 1 (NDH-1) complexes were found by screening to be the most promising candidates for disruption to improve 1,3-PDO production. The effect of disruption of the gene encoding a subunit of the NDH-1 complex was evaluated in the 1,3-PDO-producing strain.

**Results and Conclusions:**

During 20 days of incubation, the *ndhF1*-null 1,3-PDO-producing strain showed the highest titers: 4.44 mM (0.338 g/L) 1,3-PDO and 30.3 mM (2.79 g/L) glycerol. In this study, we successfully improved 1,3-PDO productivity on the basis of in silico simulation of gene disruption.

**Electronic supplementary material:**

The online version of this article (10.1186/s12934-017-0824-4) contains supplementary material, which is available to authorized users.

## Background

**Fig. 1:**
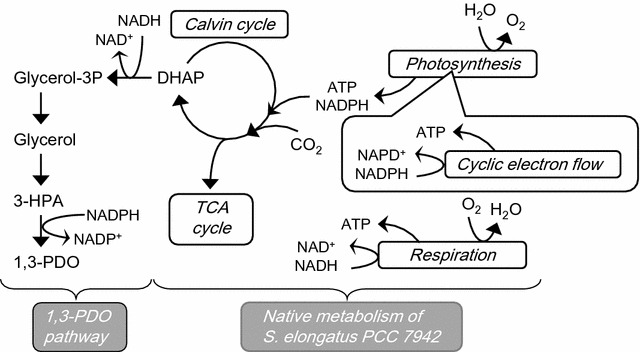
The metabolic pathway of the 1,3-PDO-producing strain

Direct production of chemicals and fuels from carbon dioxide by cyanobacteria is one of the promising technologies to reduce carbon dioxide emissions and the dependence on fossil fuels [1, 2]. Compared with biological production using heterotrophic microorganisms, biological production using cyanobacteria can avoid various complicated processes for harvesting and decomposition of biomass. The introduction of a synthetic metabolic pathway composed of genes from other organisms into cyanobacteria is a key technology for production of various chemicals directly from carbon dioxide. Some of these valuable chemicals include isobutyraldehyde, isobutanol [3, 4], ethanol [5], 2,3-butanediol [6, 7], 2-methyl-1-butanol [8], 1-butanol [9, 10], acetone [11, 12], 3-hydroxybutyrate [13], ethylene [14, 15], isoprene [16, 17], 1,2-propanediol (1,2-PDO) [18], and glycerol [19, 20]. In addition, we successfully developed *Synechococcus elongatus* PCC 7942 to produce 1,3-propanediol (1,3-PDO) by introduction of a synthetic metabolic pathway [21]. 1,3-PDO is a valuable chemical widely used for manufacture of polymers, paint, solvents, and antifreeze agents [22]. In the introduced synthetic metabolic pathway, dihydroxyacetone phosphate (DHAP) in the Calvin cycle is converted to 1,3-PDO via glycerol (Fig. 1). The strain into which the synthetic pathway was introduced was found to produce 3.79 mM (0.29 g/L) 1,3-PDO and 12.6 mM (1.16 g/L) glycerol during 14 days of incubation [21]. Although chemical production directly from carbon dioxide is a key advantage of cyanobacteria compared with other heterotrophic organisms, even the ethanol production by cyanobacteria showing the highest titer (5.5 g/L) [5] among various chemicals has not been increased over 10 g/L. Therefore, the improvements of productivity are key issues to make production by engineered cyanobacteria practical and cost effective.

In silico simulation is an powerful methodology to improve productivity with low experimental costs. The stoichiometric metabolic model not requiring kinetic parameters of metabolic reactions is comparatively applicable to analysis of whole-cell metabolism. Flux balance analysis (FBA) can predict metabolic flux distribution under constraint conditions [23]. FBA using the genome-scale metabolic model (GSM) has been applied to improvement, for example, of succinic acid [24], threonine [25], valine [26], and lycopene [27] production by *Escherichia coli*. For screening of effective candidates for gene manipulation, various strategies have been established for prediction of the effects of a gene knockout, knockdown, and overexpression [28]. Shastri and Morgan [29] were the first to construct a stoichiometric metabolic model of cyanobacteria composed of the central metabolism and photosystem of *Synechocystis* sp. PCC 6803. So far, the metabolic models of some cyanobacteria used for bioproduction (*Synechocystis* sp. PCC 6803, *Synechococcus* sp. PCC 7002, *Synechococcus elongatus* PCC 7942, *Arthrospira platensis*, *Cyanothece* sp. ATCC 51142) have been constructed on the basis of an annotated genome sequence [30–32]. Anfelt et al. [9] reported that introduction of the phosphoketolase pathway increases cellular acetyl-CoA level and butanol productivity in *Synechocystis* sp. PCC 6803. FBA was used for validation of this genetic modification to increase theoretical productivity of butanol. Furthermore, some simulations based on FBA have predicted various metabolic modifications (knockout and overexpression) to increase theoretical production by cyanobacteria, for example, of biofuels (1-butanol, 1-octanol, limonene, ethanol) and succinic acid by *Synechocystis* sp. PCC 6803 [33–35] and biofuels (butanol, ethanol) by *Synechococcus* sp. PCC 7002 [36]. Nonetheless, there are only a few reports that these predicted strategies for productivity improvement applied experimentally [35].

In this study, we applied FBA to prediction of metabolic flux distribution in a 1,3-PDO-producing strain of *S. elongatus* PCC 7942 constructed in our previous work [21]. The candidates for the gene knockout to increase 1,3-PDO productivity were estimated by FBA with the minimization of metabolic adjustment (MOMA) algorithm [37]. The positive effect of the gene knockout on 1,3-PDO productivity after screening by in silico simulation was experimentally validated in the 1,3-PDO-producing strain.

## Methods

### Chemicals and reagents

All the chemicals were purchased from Wako Pure Chemical Industries, Ltd. (Osaka, Japan) unless specified otherwise. Restriction enzymes, phosphatase (New England Biolabs; Ipswich, MA, USA), ligase (Rapid DNA Ligation Kit, Roche; Mannheim, Germany), and DNA polymerase (KOD Plus Neo DNA polymerase, TOYOBO Co., Ltd.; Osaka, Japan) were used for cloning. Oligonucleotides were synthesized by Life Technologies Japan, Ltd. (Tokyo, Japan).

### Culture media

A modified BG11 medium supplemented with 20 mM HEPES–NaOH (pH 7.5) for pH stabilization was used for cultivation of *S. elongatus* PCC 7942 (Life Technologies Corporation; Carlsbad, CA, USA). Hereafter, this medium is referred to as the BG11 medium. The composition of the BG11 medium used in this study was the same as previously reported [21]. Antibiotics (10 μg/mL kanamycin, 20 µg/mL spectinomycin, and 5 µg/mL chloramphenicol) were added to the BG11 medium when appropriate.

### Production conditions

The incubation conditions for 1,3-PDO production were the same as those described previously [21]. Cell density (OD_730_) was measured using Infinite 200 PRO (TECAN; Männedorf, Switzerland). For determination of dry cell weight (DCW), cells in culture were harvested on the Ominipore™ membrane filter (JHWP04700; Merck Millipore; Darmstadt, Germany) by filtration under reduced pressure. Cell harvest filters were dried at 65 °C for 12 h. The weight differences before and after cell harvesting were determined as DCW in culture.

### Plasmid construction

Plasmids and primers used in this study are listed in Additional file 1: Tables S1 and S2, respectively. To construct a plasmid for gene disruption, the upper and lower regions of a target gene were amplified for homologous recombination by PCR. The primer sets for the upstream region of *ndhD1*, *ndhD2*, *ndhD3*, *ndhD4*, and *ndhF1* were T2711–T2712, T3189–T3190, T2723–T2724, T2719–T2720, and T3208–3209, respectively. The primer sets for the downstream region of *ndhD1*, *ndhD2*, *ndhD3*, *ndhD4*, and *ndhF1* were T3187–T3188, T3191–T3192, T2787–T2726, T2721–2722, and T2713–T2714, respectively. The amplified upstream and downstream regions except for *ndhF1* were digested by *Kpn*I–*Hin*dIII and *Hin*dIII–*Bam*HI, respectively, and ligated into *Kpn*I–*Bam*HI sites of pTA703. Only for *ndhF1*, the upstream and downstream regions were digested with *Sal*I–*Hin*dIII and *Hin*dIII–*Bam*HI, respectively, and ligated into *Sal*I–*Bam*HI sites of pTA703. A chloramphenicol resistance cassette was amplified from pZA31-*luc* (Expressys; Ruelzheim, Germany) by PCR using primers T2128–T2129. The amplified fragment digested with *Hin*dIII was ligated into the *Hin*dIII site of each plasmid harboring upstream and downstream regions of a target gene, thus creating plasmids pTA1811 (*ndhD1* disruption), pTA1812 (*ndhD2* disruption), pTA1641 (*ndhD3* disruption), pTA1640 (*ndhD4* disruption), and pTA1828 (*ndhF1* disruption).

### Strain construction

The constructed plasmids (pTA1811, 1812, 1641, 1640, and 1828) were used for gene disruption in TA1297 (wild-type strain), TA2984 (1,3-PDO-producing strain), and TA3800 (glycerol-producing strain). TA2984 and TA3800 were constructed in our previous studies [21, 38]. The strains used in this study are listed in Additional file 1: Table S1.

### Product analysis

Culture supernatants were centrifuged (20,000×*g*, 10 min) and filtered using Minisart RC4 (Sartorius; Goettingen, Germany). Concentrations of glycerol and 1,3-PDO in filtered samples were determined using a high-performance liquid chromatograph (LC-20AD, Shimadzu; Kyoto, Japan) equipped with an autosampler (AOC-20; Shimadzu), a SUGAR SP-G guard column (Shodex; Tokyo, Japan), a SUGAR SP0810 column (Shodex), and a refractive index detector (RID-10A; Shimadzu). Operating conditions were as follows: injection volume, 5 µL; mobile phase, MilliQ water; flow rate, 1.0 mL/min in isocratic mode; column temperature, 80 °C.

### In silico simulation of metabolic flux distribution

#### FBA for TA2984 (1,3-PDO-producing strain)

The stoichiometric metabolic model based on the annotated genome sequence of *S. elongatus* PCC 7942 published by Knoop [39] (hereafter called GSM_7942 in this study) served as a basic metabolic model of the wild-type strain in this study. GSM_7942 is composed of 595 genes, 666 reactions, and 533 metabolites. The information on the introduced synthetic metabolic pathway for 1,3-PDO production (8 genes, 6 reactions, and 3 metabolites) was added to GSM_7942, resulting in a metabolic model of TA2984 (hereafter called GSM_7942_PDO in this study). GSM_7942_PDO is composed of 603 genes, 672 reactions, and 536 metabolites. GSM_7942_PDO was used to perform FBA using the COBRA toolbox 2.05 [40] on Matlab (Mathworks Inc., Natick, MA).

#### Gene disruption simulation in TA2984 with the MOMA algorithm

This simulation was performed on GSM_7942_PDO with scripts provided in COBRA toolbox 2.05 using Gurobi 6.03 (Gurobi optimization Inc., USA) as a solver.

### Respiratory and photosynthetic activities

Chlorophyll concentrations were measured by extraction in methanol [41]. Oxygen consumption (respiration) and evolution (photosynthesis) from whole cells were measured with a Clark-type electrode (OXYT-1, Hansatech Instruments, Norfolk, UK) at a chlorophyll concentration of 10 µg/mL. The temperature of the electrode chamber was maintained at 30°C, and the chamber contents were continuously stirred. Saturated light (1000 μE/m^2^/s) and growth light (100 μE/m^2^/s) provided by a digital LED source (PD3-5024-4-EI, CCS, Kyoto, Japan) were used for measurement of photosynthetic activity. Photon flux density was measured with IKS-27 (Koito; Tokyo, Japan). Respiratory activity was similarly measured in complete darkness.

### Measurement of ATP content

ATP extraction and measurement were basically performed by the method of Wang et al. [42]. The appropriate number of cells at growth phase (corresponding to 1-mL culture at OD_730_ of 1.0) was collected, and ATP was extracted from pelleted cells by resuspension in a 1% (w/v) trichloroacetic acid solution. After centrifugation at 20,000×*g* for 10 min at 4 °C, the supernatant was neutralized with 1 M Tris–acetate buffer (pH 7.8). The concentration of ATP in the neutralized sample was determined using the ATP determination kit (A22066, Molecular Probes Inc., Oregon, USA).

### Measurement of intracellular metabolites

Intracellular metabolites of TA2984 (1,3-PDO-producing strain) and TA4058 (*ndhF1*-null 1,3-PDO-producing strain) were measured by LC-QqQ-MS and GC-Q-MS analyses. Sample preparation and analysis were performed by the method described by Soma and Hanai [43].

### Measurement of glycogen content

Glycogen extraction was basically performed by the method of Suzuki et al. [44]. Cells at early stationary phase (days 10) were used for glycogen measurement. The extracted glycogen was mixed with glucoamylase (Oriental Yeast Co., Ltd.; Tokyo, Japan) in 2.5 mM Na-acetate (pH 5.0) and incubated at 40 °C for 1 h. After centrifugation, glucose generated from glycogen was measured using BF-7 (Oji Scientific Instruments, Hyogo, Japan) equipping glucose electrode (ED07-0003, Oji Scientific Instruments).

## Results and discussion

### FBA of the 1,3-PDO-producing strain

We previously constructed a 1,3-PDO-producing strain, designated as TA2984, by introduction of a synthetic metabolic pathway. A four-step reaction of the introduced pathway converts DHAP, one of the metabolites in the Calvin cycle, into 1,3-PDO via glycerol (Fig. 1). Construction of the stoichiometric metabolic model based on the annotated genome sequence of *S. elongatus* PCC 7942 was first reported by Triana et al. [31]. In the same year, the comparable scale of a metabolic model of this strain was published by Knoop et al. [39]. We used Knoop’s model composed of 595 genes, 666 reactions, and 533 metabolites as a basic metabolic model of the wild-type strain of *S. elongatus* PCC 7942 (hereafter called GSM_7942 in this study). The additional information on the introduced synthetic metabolic pathway for 1,3-PDO production (8 genes, 6 reactions, and 3 metabolites) was added to GSM_7942, resulting in a metabolic model of the 1,3-PDO-producing strain composed of 603 genes, 672 reactions, and 536 metabolites (hereafter called GSM_7942_PDO in this study).

**Fig. 2:**
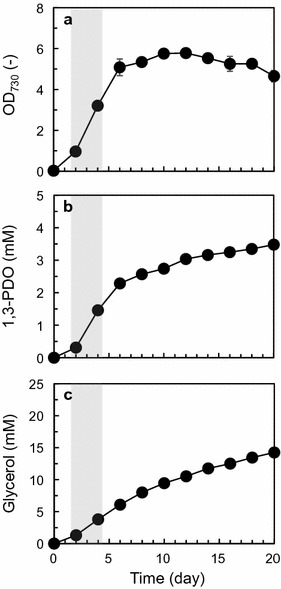
1,3-PDO production by strain TA2984, into which the synthetic metabolic pathway for 1,3-PDO production was introduced. **a** Growth, **b** 1,3-PDO production, **c** glycerol production. The exponential growth phase (days 2–4) in the shadowed area is applied to FBA. Data are presented as mean ± SD of three individual experiments

**Fig. 3:**
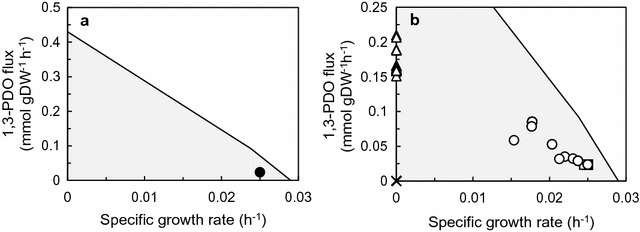
FBA for 1,3-PDO production. **a** Two-dimensional solution space of 1,3-PDO production and cell growth is presented as a shadowed area. Experimental data from Fig. 2 are plotted as filled circles. **b** The metabolic flux distributions observed in gene disruption simulation are plotted in solution space. Circles, squares, triangles, and crosses represent four groups of the simulated phenotypes. Circles (36) and squares (231) are groups indicating higher and lower productivity relative to the experimental data, respectively. Triangles (254) and crosses (74) are groups indicating a null value of a specific growth rate and no solution satisfying constraint conditions

GSM_7942_PDO was used for prediction of metabolic flux distribution of TA2984 into which the synthetic metabolic pathway for 1,3-PDO was introduced [21]. To predict metabolic flux distribution using a stoichiometric metabolic model, input fluxes to the cell and output fluxes from the cell were needed. For TA2984, the former represents the carbon fixation rate and photon uptake rate, and the latter represents the biomass production rate (corresponding to the specific growth rate) and specific production rates of 1,3-PDO and glycerol. To determine output fluxes, TA2984 was incubated under the same conditions as in our previous report [21]. As a result, TA2984 produced 3.48 mM (0.265 g/L) 1,3-PDO and 14.3 mM (1.32 g/L) glycerol directly from carbon dioxide during 20 days of incubation (Fig. 2). Although the output fluxes were observed in the experimental data shown in Fig. 2, it was difficult to experimentally determine the input fluxes (carbon fixation rate and photon uptake rate). Therefore, GSM_7942_PDO was used for calculation of the input fluxes capable of satisfying the output fluxes observed experimentally. Because the assumption of a steady state of metabolism is one of the key constraints in FBA [23], the data from the exponential growth phase (days 2–4) were applied to the prediction. First, the specific growth rate and the specific production rates of 1,3-PDO and glycerol in the exponential growth phase were calculated to be 0.0250/h, 0.0273 mmol/(g DCW)/h, and 0.0515 mmol/(g DCW)/h, respectively. Next, the input fluxes of carbon fixation and photon use were calculated and found to be 1.35 and 19.0 mmol/(g DCW)/h, respectively. These input fluxes were calculated by the method of two-step optimization strategy of Shastri and Morgan [29] with some modifications. These calculated input fluxes and experimentally obtained output fluxes were used for prediction of the metabolic flux distribution of TA2984 in the exponential growth phase. Under the conditions restricted by input fluxes of carbon fixation and photon use, the maximized specific growth rate corresponded to 0.0290/h without 1,3-PDO and glycerol production. A two-dimensional solution space between the specific growth rate and the specific production rate of 1,3-PDO was then described (Fig. 3a). The experimental data shown in Fig. 2 were plotted in the described solution space.

### Simulation of gene disruption in TA2984 (1,3-PDO-producing strain)

The metabolic flux distribution of TA2984 using FBA was applied to the gene disruption simulation based on the MOMA algorithm [37]. This is one of the algorithms used for simulation of sequential gene deletion assuming that the cell adapts to a mutation to minimize the changes of metabolic flux distribution by the gene deletion. The estimated candidates for gene deletion based on this algorithm successfully increased lycopene production in *E. coli* [45]. Because the objective of our simulation was to screen promising candidates for gene disruption for improvement of 1,3-PDO productivity, we set the specific production rate of 1,3-PDO obtained experimentally [0.0250 mmol/(g DCW)/h] as the lower limit. The metabolic flux distribution corresponding to each of the 595 gene disruptions in GSM_7942_PDO except for the 8 genes of 1,3-PDO producing synthetic metabolic pathway was simulated and the simulated results on 581 strains with single-gene disruption were plotted according to the estimated specific growth rate and estimated specific production rate of 1,3-PDO (Fig. 3b). The simulated results on the 581 strains with single-gene disruption were roughly subdivided into four groups. These four groups are presented as different symbols in Fig. 3b.

The first group (crosses in the figure) was composed of 74 strains that could not satisfy the constraint conditions and lower limit of 1,3-PDO production. The second group (triangles) included 254 strains showing a higher specific production rate of 1,3-PDO, but a null value of the specific growth rate. This may also mean that each gene disrupted in the 254 strains was essential for growth. It was likely that the disruption of any of the 328 genes classified into these two groups was not applicable to improvement of 1,3-PDO productivity. The metabolic flux distributions of the other 267 strains with single-gene disruption could show a certain specific growth rate and higher specific production rate of 1,3-PDO as compared with the experimental data. We screened the 267 candidates for more desirable ones using the product of the specific growth rate and specific production rate as an indicator. Out of the 267 candidates, 231 showed a lower product of the specific growth rate and specific production rate and were classified into the third group (squares).

Finally, 36 genes were classified into the fourth group (circles) as promising candidates to be disrupted for increasing 1,3-PDO productivity (Additional file 1: Table S3). Only 10 patterns of metabolic flux distribution were obtained from 36 strains in the fourth group. First, in GSM_7942, the annotated-gene information was defined by a gene–protein reaction (GPR) association [46]. Under the definition of the GPR association, each disruption of a subunit of a protein complex indicates the same flux distribution. In this study, 22 genes involved in NADPH dehydrogenase-1 (NDH-1) complexes and four genes involved in cytochrome *c* oxidase were classified into the fourth group. Second, two cases indicating the same metabolic flux distribution were observed in different two-gene disruptions (Synpcc7942_0191 and 1501, and Synpcc7942_2079 and 2080). These genes were annotated as genes encoding an enzyme catalyzing a serial reaction without any blanching pathway. The other six metabolic flux distributions were examined by single-gene disruption. Based on the value of the product of the specific growth rate and specific production, the disruption of 22 genes involved in the NDH-1 complex was found by screening as the most promising candidate for improvement of 1,3-PDO production among the 10 patterns of the fourth group.

### Disruption of genes involved in NDH-1 complexes

**Fig. 4:**
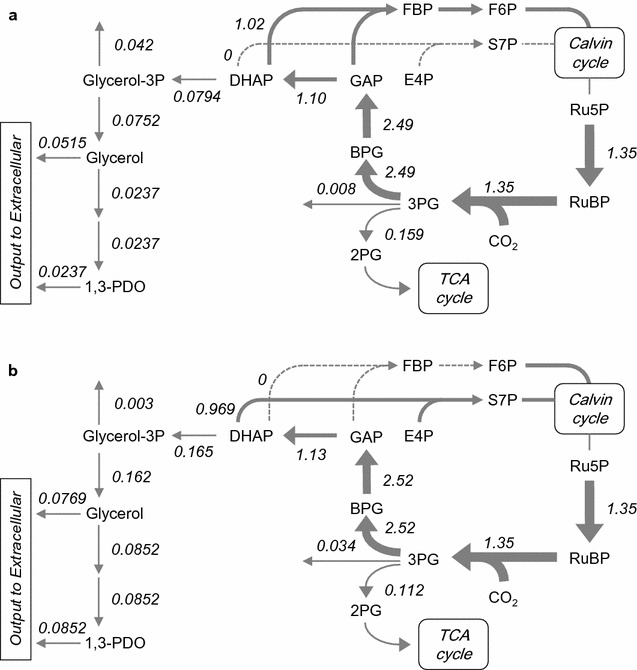
Metabolic flux distribution around the 1,3-PDO-producing pathway. **a** Estimated data on strain TA2984 (parental strain for 1,3-PDO production) from experimental data of Fig. 2. **b** Simulation data on NDH-1 elimination based on the MOMA algorithm. Arrows and their thickness represent reactions and the scale of flux, respectively. The tilted values near arrows represent estimated flux [mmol/(g DCW)/h]

Simulated metabolic flux distribution for each strain by gene disruption indicated that the elimination of the NDH-1 complex is the most effective candidate for improvement of 1,3-PDO productivity. The estimated metabolic flux distributions with and without NDH-1 complex elimination were compared (Fig. 4). Each value with an arrow represents the flux [mmol/(g DCW)/h] calculated from experimental data (Fig. 4a) and predicted one of the NDH-1 complex-null strains using the MOMA algorithm (Fig. 4b). In all simulations of gene disruption, the flux of carbon fixation [1.35 mmol/(g DCW)/h] was assumed to not be changed by any gene disruptions. The stoichiometry of ribulose-bisphosphate carboxylase/oxygenase (RubisCO) reaction in GSM_7942 means that 1.97 molecules of 3-phosphoglycerate (3-PG) were produced from 1 molecule of ribulose-1,5-bisphosphate (RuBP), considering photorespiration [39]. Therefore, the flux of 3-phosphoglycerate (3-PG) production corresponded to 2.66 mmol/(g DCW)/h. By means of elimination of the NDH-1 complex, some metabolic fluxes around the 1,3-PDO-producing pathway were predicted to be changed. First, the blanching ratio of the 3-PG-consuming flux into the glycolysis (2-phosphoglycerate; 2-PG) and the Calvin cycle (1,3-bisphosphoglycerate; BPG) was changed. NDH-1 complex elimination increased the flux of fixed carbon into the Calvin cycle. Second, each flux of DHAP production from GAP (glyceraldehyde 3-phosphate) and that of DHAP consumption for S7P (sedoheptulose 7-phosphate) or FBP (fructose-1,6-bisphosphate) synthesis was increased and decreased, respectively, resulting in the increase in the flux into the 1,3-PDO-producing pathway.

NDH-1 complexes in cyanobacteria are involved in several cellular functions (respiration, cyclic electron flow, and carbon dioxide acquisition) and are subdivided into four variants (NDH-1L, L′, MS, and MS′) by function and subunit composition according to reverse-genetic studies [47]. The phenotypic analyses of gene knockout mutants revealed the functional differences among NDH-1 complexes, indicating that NDH-1L and L′ are involved in respiration and cyclic electron flow, whereas NDH-1MS and MS′ participate in carbon dioxide acquisition [48, 49]. Because NdhB is one of the common subunits for all four variants, it was expected that *ndhB* disruption would be desirable to represent the metabolic flux distribution observed in the gene disruption simulation (Fig. 4b, Additional file 1: Table S3). Nonetheless, all functions of NDH-1 complexes were drastically decreased in the *ndhB*-null mutant, in particular, this mutant could not grow under ambient air conditions [50–52]. Indeed, *ndhB* could not be completely disrupted in the wild-type strain and TA2984 (1,3-PDO-producing strain) in our experiment. On the other hand, it was reported that four mutants in which each gene encoding the unique subunit in four NDH-1 complexes is disrupted can grow under photoautotrophic and ambient air conditions [48]. Therefore, we tried to disrupt the genes encoding these unique subunits in TA2984.

In contrast to the strain with *ndhB* disruption, the gene knockout strains of *ndhD1* (Synpcc7942_1976, unique subunit gene for NDH-1L), *ndhD2* (Synpcc7942_1439, that for NDH-1L′), *ndhD3* (Synpcc7942_2092, that for NDH-MS), and *ndhD4* (Synpcc7942_0609, that for NDH-MS′) based on the 1,3-PDO-producing strain (TA2984) were successfully constructed (Additional file 1: Table S1). These four strains were applied to 1,3-PDO production (Additional file 2: Figure S1). All the strains showed a growth defect as compared with TA2984, and the growth defect was more severe in three strains except for TA3672 (*ndhD3*-null strain; Additional file 2: Figure S1A). Among the four strains, TA4021 (*ndhD1*-null strain) showed the highest titers of 1,3-PDO (4.04 mM, 0.307 g/L) and glycerol (19.6 mM, 1.81 g/L) during 20 days of incubation (Additional file 2: Figure S1B, C). These titers of 1,3-PDO and glycerol shown by TA4021 were 1.16- and 1.38-fold higher than those of TA2984 (Fig. 2). Table 1 shows output fluxes (specific production rates or specific growth rates) for the wild type and the knockout strains observed in the exponential growth phase (days 2–4) and those calculated by the simulation. This strain revealed a higher specific production rate of 1,3-PDO in the exponential growth phase than TA2984 did (Table 1). The titer of 1,3-PDO produced by TA4022 (*ndhD2*-null strain) did not increase (Additional file 2: Figure S1B) while a significantly improved production rate of 1,3-PDO was observed in this strain (Table 1). The specific production rate of 1,3-PDO by TA3672 (*ndhD3*-null strain) was the lowest among the four gene knockout strains and TA2984 (Table 1). On the other hand, TA3671 (*ndhD4*-null strain) showed the highest production rate of 1,3-PDO (Table 1), but the titer of 1,3-PDO produced by this strain was lower only than the titer of TA2984 (Additional file 2: Figure S1).

**Table 1:** Output fluxes observed by experiments (days 2–4) and the simulated output fluxes

Experimental or simulated data	Biomass	1,3-PDO	Glycerol	Sum of products 1,3-PDO + glycerol	1,3-PDO ratio 1,3-PDO/sum of products
TA2984 (parental strain for 1,3-PDO production)	0.0250	0.0237	0.0515	0.0752 (1.00)	0.315 (1.00)
Simulated data of NDH-1 elimination	0.0177	0.0852	0.0769	0.162 (2.15)	0.526 (1.67)
TA4021 (*ndhD1*-null strain of TA2984)	0.0292	0.0319	0.0835	0.115 (1.53)	0.277 (0.88)
TA4022 (*ndhD2*-null strain of TA2984)	0.0296	0.0299	0.0750	0.105 (1.40)	0.285 (0.90)
TA3672 (*ndhD3*-null strain of TA2984)	0.0290	0.0213	0.0562	0.0775 (1.03)	0.275 (0.87)
TA3671 (*ndhD4*-null strain of TA2984)	0.0371	0.0382	0.113	0.1512 (2.01)	0.253 (0.80)
TA4058 (*ndhF1*-null strain of TA2984)	0.0258	0.0497	0.163	0.213 (2.83)	0.233 (0.74)

**Fig. 5:**
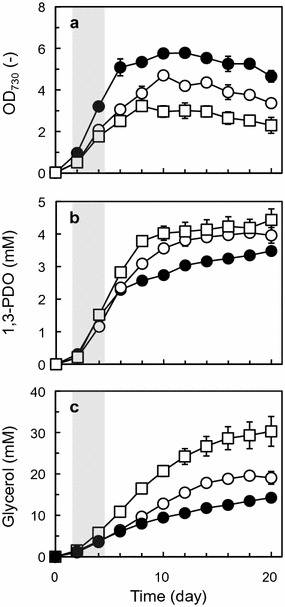
1,3-PDO production by a gene knockout mutant. **a** Growth, **b** 1,3-PDO production, **c** glycerol production. Filled circles, open circles, and open squares represent data on TA2984 (parental strain for 1,3-PDO production), TA4021 (*ndhD1*-null strain), and TA4058 (*ndhF1*-null strain), respectively. Data are presented as mean ± SD of three individual experiments

NdhF1 is a unique subunit commonly present in two NDH-1 complexes: NDH-1L and L′ [47]. It was expected that *ndhF1* disruption would lead to the additive phenotype of *ndhD1* and *ndhD2* knockouts. Other studies on gene knockout mutants of *ndhD1* and *ndhF1* showed that the NDH-1L complex is missing in each mutant [53, 54]. Just as *ndhD1*-*D4*, the *ndhF1*-null 1,3-PDO-producing strain designated as TA4058 was successfully constructed (Additional file 1: Table S1). The growth of TA4058 was more defective than that of TA4021 and TA4022, indicating that the *ndhF1* disruption affects cellular function more as compared with each single-gene disruption of *ndhD1* or *ndhD2* (Fig. 5a and Additional file 2: Figure S1A). During 20 days of incubation, the titers of 1,3-PDO and glycerol produced by TA4058 corresponded to 4.44 mM (0.338 g/L) and 30.3 mM (2.79 g/L), respectively (Fig. 5b, c). Both titers were the highest among the other gene knockout strains and the parental strain for 1,3-PDO production. These results indicated that the disruption of NDH-1 complexes (NDH-1L and L′), which have a function in respiration and cyclic electron flow, positively affected 1,3-PDO and glycerol production. The specific production rate of 1,3-PDO by TA4058 in the exponential growth phase (days 2–4) was significantly enhanced and the highest among all the constructed strains (Table 1). Unfortunately, this value [0.0497 mmol/(g DCW)/h] was inferior to that of simulation of the NDH-1 complex elimination [0.0852 mmol/(g DCW)/h]. Nonetheless, the sum of the specific production rates of 1,3-PDO and glycerol meaning the utilization rate of DHAP for the 1,3-PDO-producing pathway in TA4058 [0.213 mmol/(g DCW)/h] was surprisingly higher than that in simulation [0.162 mmol/(g DCW)/h]. The strain with higher total DHAP utilization showed a lower ratio of 1,3-PDO production to total DHAP utilization, indicating that the later part of the 1,3-PDO-producing pathway converting glycerol to 1,3-PDO is the bottleneck of effective production (Table 1). This result is consistent with findings of our recent study on 1,3-PDO production improvement by promoter exchange in the later part of the 1,3-PDO producing pathway [38]. It is expected that the strategy involving a combination of promoter exchange and NDH-1 complex disruption is a promising method for further improvement of 1,3-PDO productivity.

Previously, we have constructed a glycerol-producing strain (TA3800) into which only the pathway converting DHAP to glycerol was introduced [38]. The effect of *ndhF1* disruption was also evaluated in TA3800. As in the 1,3-PDO producing strain, the *ndhF1* disruption resulted in a growth defect and improvement of the production titer (Additional file 2: Figure S2). The growth defect caused by the *ndhF1* disruption in the 1,3-PDO-producing strain was more significant than that in the wild-type strain and glycerol-producing strain (Additional file 2: Figure S2A–C). The highest titer of glycerol produced by the *ndhF1*-null glycerol-producing strain designated as TA4059 reached 36.7 mM (3.38 g/L; Additional file 2: Figure S2D). This value was almost equal to the sum of the titers of 1,3-PDO and glycerol (34.7 mM) shown by TA4058 (Fig. 5) and about twofold higher than that of TA3800 (Additional file 2: Figure S2E). Although the reason for the significant growth defect caused by *ndhF1* disruption in the 1,3-PDO-producing strain was not clear, glycerol production was successfully stimulated by the *ndhF1* disruption, as was 1,3-PDO production. The titer of glycerol produced by TA4059 was the highest among other chemicals (glycerol, 1,3-PDO, and 1,2-PDO) produced from DHAP by engineered cyanobacteria [18–20, 38] and this titer corresponded to high titer compared to other chemicals produced not from DHAP by engineered cyanobacteria, such as ethanol [5], sucrose [55], 2,3-butanediol [7], and lactate [56]. Although various chemicals have been produced by engineered cyanobacteria as summarized in some reviews [1, 2], the research resulting from the titer of product over 3 g/L directly from carbon dioxide seems to be still rare.

### Metabolism changes caused by ndhF1 disruption

The disruption of *ndhF1* successfully increased metabolic flux of DHAP into the 1,3-PDO-producing pathway as expected on the basis of the in silico simulation (Table 1) and improved the titers of 1,3-PDO and glycerol production (Fig. 5 and Additional file 2: Figure S2). This result suggests that in silico simulation is an effective method for metabolic improvement of bioproduction in cyanobacteria. To identify the causes of improved productivity, the metabolic changes caused by the *ndhF1* disruption were analyzed. It was expected that the *ndhF1* disruption affected to respiration and cyclic electron flow [47]. Therefore, at first, the respiration activities of TA4058 (*ndhF1*-null 1,3-PDO-producing strain) and TA2984 (parental strain) were measured. As the result, TA4058 showed lower respiratory activity in the dark and almost the same photosynthetic activity under saturated light conditions (1000 μE/m^2^/s) as compared with TA2984 (Additional file 2: Figure S3). This finding was consistent with the phenotype of a *ndhF1*-null strain of *Synechococcus* sp. PCC 7002 observed in another study [57]. Under growth light conditions (100 μE/m^2^/s), the photosynthetic activity of TA4058 was lower than that of TA2984, being consistent with the growth defect of TA4058 (Fig. 5).

The physiological role of cyclic electron flow is known to increase the ATP/NADPH ratio produced by photosynthetic electron flow [58]. So, next, ATP contents in TA4058 and TA2984 were measured. TA4058 showed lower cellular ATP content as compared with TA2984 (Additional file 2: Figure S4). In the respiratory chain, the reducing power (NADH and FADH_2_) mainly produced by glycolysis and the TCA cycle is used for generating a proton gradient and H_2_O from O_2_. The generated proton gradient functions as a driving force of ATP synthase for ATP production. Whole or partial loss of cyclic electron flow and respiratory activity under the influence of the *ndhF1* disruption may decrease cellular ATP content.

The intracellular metabolites involved in a reaction with ATP seemed to reflect the ATP deficiency in TA4058. For example, ATP is consumed in reactions producing RuBP and ADP-glucose, and is produced in the reaction producing pyruvate. In ATP-consuming reactions, substrates (ribulose-5-phosphate: Ru5P and glucose-1-phosphate: G1P) and products (RuBP and ADP-glucose) were detected at high and low concentrations, respectively. Conversely, lower concentration of a substrate (phosphoenolpyruvate: PEP) and higher concentration of a product (pyruvate) were observed in an ATP-producing reaction. In particular, the metabolites around the glycogen synthesis pathway seemed to be affected by the ATP deficiency. Fructose-6-phosphate (F6P) is a metabolite of the branching point for glycogen synthesis from the Calvin cycle and is sequentially converted to glucose-6-phosphate (G6P), glucose-1-phosphate (G1P), and ADP-glucose. In this glycogen synthesis pathway, significant accumulation (more than fivefold) was observed for metabolites except for ADP-glucose produced with ATP consumption. This result suggests that glycogen synthesis is repressed in TA4058 caused by ATP deficiency. Because other studies showed that higher accumulation of glycogen is observed in stationary phase [44, 59], the glycogen concentrations of TA2984 and TA4058 were measured at 10 days (Additional file 2: Figure S6). As expected, the intracellular glycogen concentration of TA4058 was about half of that of TA2984 (Additional file 2: Figure S6A).

It is well known that the respiratory activity is strongly related to catabolic metabolism (glycolysis and TCA cycle) in heterotrophic organisms. We hypothesized that the repressed respiratory activity can decrease the metabolic flux in catabolic metabolism generating reducing power in photosynthetic organisms, too. The decreasing of 3-PG-consuming flux into glycolysis by the *ndhF1* disruption in the simulation result (Fig. 4) was consistent with the hypothesis. Quantification of intracellular metabolites also supported this hypothesis (Additional file 2: Figure S5). Most of the metabolites in the Calvin cycle showed higher intracellular concentration in TA4058 than in TA2984. On the other hand, the metabolites in the glycolysis and TCA cycle were mostly lower in TA4058 compared with TA2984.

Not only greater flux of 3-PG into the Calvin cycle but also repression of glycogen synthesis may increase the productivity values of 1,3-PDO and glycerol. These results indicate that the productivity improvement was caused by the metabolism adapted to energy imbalance induced by the *ndhF1* disruption. Hasunuma et al. [60] successfully improved the carbon fixation rate in *Synechocystis* sp. PCC 6803 by overexpression of the *flv3* gene involved in NADPH consumption. The strategy of acceleration of NADPH consumption also showed the energy balance to change cellular metabolism of cyanobacteria. In the present study, the metabolism changes intended to adapt to the energy imbalance (ATP deficiency) were applied to chemical production (1,3-PDO and glycerol). Taking into consideration of the points mentioned above, *ndhF1* disruption is a good strategy for enhancement of production of other chemicals (for instance, 1,2-PDO and sucrose) from metabolites in the Calvin cycle in cyanobacteria [18, 38, 55].

The simulation result of the metabolic flux distribution of the *ndhF1*-null and wild type strain (Fig. 4) showed the 3-PG-consuming flux to 2-PG or TCA cycle was decreased by the *ndhF1* disruption. This result suggested that the *ndhF1*-null strain was not suitable for the chemical productions from pyruvate or acetyl-CoA. Yoshikawa et al. [35], however, showed the *ndhF1*-null PCC6803 strain with ethanol producing pathway was produced 145% more ethanol (0.132 g/L) from pyruvate than the wild type strain with the same pathway (0.191 g/L). It is difficult to explain clearly the reason why the suggestion based on our simulation result is different from their result, but one possible reason would be the driving force by ATP generation by the reaction from PEP to pyruvate to compensate the ATP deficiency in the *ndhF1*-null strain.

Recently, Broddrick et al. [61] constructed GEM of metabolism in PCC7942 composed of 785 genes. This model was constructed by using the information about essential genes revealed by the random barcode transposon site sequencing and physiological data for photoautotrophic metabolism. Using this model or the larger models than that we used, there are possibilities to screen out the other candidate genes for knockout to improve 1,3-PDO productivity.

## Conclusions

In this study, the productivity of 1,3-PDO and glycerol in *S. elongatus* PCC 7942 were successfully improved by gene manipulation based on in silico simulation of gene disruptions. The best candidate for improvement of 1,3-PDO productivity was found to be the elimination of NDH-1 complexes involved in several cellular functions: respiration, cyclic electron flow, and carbon dioxide uptake (Additional file 1: Table S3). Genes encoding NDH-1 complexes were actually disrupted in TA2984 (parental strain for 1,3-PDO production). The effective improvement of productivity was achieved by disruption of the NDH-1 complexes that are related to respiration and cyclic electron flow (Additional file 2: Figure S1). Finally, TA4058 (*ndhF1*-null 1,3-PDO-producing strain) was found to produce 1.28-fold more 1,3-PDO (4.44 mM, 0.338 g/L) and 2.13-fold more glycerol (30.3 mM, 2.79 g/L) than TA2984 did (Fig. 5). The metabolism adapted to the energy imbalance caused by the *ndhF1* disruption may enhance the 3-PG-consuming flux into the Calvin cycle and can be suitable for 1,3-PDO and glycerol production (Additional file 2: Figure S5). This result opens up exciting opportunities for applying in silico simulation to metabolic improvement of cyanobacteria and is suggestive of the feasibility of metabolic changes focused on the energy balance.

## Additional files

**Additional file 1.** Additional tables.

**Additional file 2.** Additional figures.
