# Supplementary material for: Metabolic engineering of Synechococcus elongatus PCC 7942 for improvement of 1,3-propanediol and glycerol production based on in silico simulation of metabolic flux distribution
Source: Microb Cell Fact. 2017 Nov 25;16:212. doi: 10.1186/s12934-017-0824-4 (PMC5702090; doi:10.1186/s12934-017-0824-4)
Supplement: Supplementary file 1 — Additional file 1. Additional tables. [file 12934_2017_824_MOESM1_ESM.docx]

Table S1. Plasmids and strains used in this study

| **Plasmid/Strain** | **Genotype** | **Reference** |
| --- | --- | --- |
| ***Plasmid*** |  |  |
| pZE22-MCS | *P*_L_lacO1:: MCS, *kan^r^* | Expressys (Ruelzheim, Germany) |
| pZE12-MCS | *P*_L_lacO1:: MCS, *amp*^r^ | Expressys (Ruelzheim, Germany) |
| pZA31-*luc* | *P*_L_lacO1:: MCS, *cm*^r^ | Expressys (Ruelzheim, Germany) |
| pTA1811 | plasmid for *ndhD1* disruption | This study |
| pTA1812 | plasmid for *ndhD2* disruption | This study |
| pTA1641 | plasmid for *ndhD3* disruption | This study |
| pTA1640 | plasmid for *ndhD4* disruption | This study |
| pTA1828 | plasmid for *ndhF1* disruption | This study |
| ***Strain*** |  |  |
| TA1297 | wild type strain of *S. elongatus* PCC 7942 | Invitrogen (CA, USA) |
| TA4057 | Δ*ndhF1* of TA1297 genome | This study |
| TA2984 | *P*_L_lacO1::*dhaB1-dhaB2-dhaB3-gdrA-gdrB-yqhD, P*lacIq::*lacI* integrated in NS I,  *P*_L_lacO1::*gpd1-hor2* integrated in NS II of TA1297 genome | Hirokawa *et al*., 2016 |
| TA4021 | Δ*ndhD1* of TA2984 genome | This study |
| TA4022 | Δ*ndhD2* of TA2984 genome | This study |
| TA3672 | Δ*ndhD3* of TA2984 genome | This study |
| TA3671 | Δ*ndhD4* of TA2984 genome | This study |
| TA4058 | Δ*ndhF1* of TA2984 genome | This study |
| TA3800 | *P*_L_lacO1::*gpd1-hor2, P*lacIq::*lacI* integrated in NS I of TA1297 genome | Hirokawa *et al.,* 2017 |
| TA4059 | Δ*ndhF1* of TA3800 genome | This study |

Table S2. Primers used in this study

| **Primer** | **Sequence** | |
| --- | --- | --- |
| T2128 | 5’ | GCCATCAAGCTTGCTTGGATTCTCACCAATAAAAAACGCCCG |
| T2129 | 5’ | GCCATCAAGCTTGATATCTGGCGAAAATGAGACGTTGATCG |
| T2711 | 5’ | GCCATCGGTACCAGAGACAGCCAAACGCGACAGC |
| T2712 | 5’ | GCCATCAAGCTTGCCATGTGGACGACCTGCG |
| T2713 | 5’ | GCCATCAAGCTTGCCTTGGCGGGAATGCAG |
| T2714 | 5’ | GCCATCGGATCCCACAGGCAACTGCTG |
| T2719 | 5’ | GCCATCGGTACCATGCTCAGTGCCCTGATTTGGC |
| T2720 | 5’ | GCCATCAAGCTTGCCGTGTAGATCAGGAATTTCGTG |
| T2721 | 5’ | GCCATCAAGCTTGCCACGGCTTGATTTCGGC |
| T2722 | 5’ | GCCATCGGATCCCTAGGTCAGAGCATCCAAGGCCC |
| T2723 | 5’ | GCCATCGGTACCATGATGTTGGTGCTTTTAATTACAGCGA |
| T2724 | 5’ | GCCATCAAGCTTGAAATGGCCGTGAAAATCAGGAAG |
| T2726 | 5’ | GCCATCAAGCTTTGTTCCAGCTTGTCGGCACG |
| T2787 | 5’ | GCCATCGGATCCTTACTGCAGGACGGCGGACAA |
| T3187 | 5’ | GCCATCAAGCTTGCATACTGACGGCGTTTGCCG |
| T3188 | 5’ | GCCATCGGATCCATGGAGATCGGGACGTTTCCCT |
| T3189 | 5’ | GCCATCGGTACCTCAGTCGATCACAGGCGGAGCAGTCATCGA |
| T3190 | 5’ | GCCATCAAGCTTTTGCCAAGCAAATGCCGATTAC |
| T3191 | 5’ | GCCATCAAGCTTAAGATGAATAAGGAGGCTAAGGCAGTGTAA |
| T3192 | 5’ | GCCATCGGATCCCTGCCGATCTTGGCAGCGC |
| T3208 | 5’ | GCCATCAAGCTTTGGCGATTCTAGCGGTGCCTT |
| T3209 | 5’ | GCCATCGTCGACCTAGGTCACCCCGAAGGCCAC |

Table S3. 36 candidates of knockout gene for improvement of 1,3-PDO productivity

| **Gene ID (Gene name)** | **Function, Activity** | **Biomass** | **1,3-PDO** | **Biomass**  **×1,3-PDO** | **Glycerol** | **Essentiality**  **(Rubin *et al*., 2015)** |
| --- | --- | --- | --- | --- | --- | --- |
| Experimental data of TA2984 (Fig. 2) | | 0.0250 | 0.0237 | 5.93×10^-4^ | 0.0515 | - |
| NDH-1 genes (22 genes)  (detailed in Table S4) | respiratory, cyclic electron flow, and  bicarbonate uptake | 0.0177 | 0.0852 | 1.51×10^-3^ | 0.132 | (detailed in Table S4) |
| Synpcc7942_0191  Synpcc7942_1501 (*serA*) | serine-glyoxylate transaminase  phosphoglycerate dehydrogenase | 0.0177 | 0.0786 | 1.39×10^-3^ | 0.0972 | essential  (two genes) |
| Synpcc7942_2079 (*ackA*)  Synpcc7942_2080 (*xfp*) | acetate kinase  phosphoketolase | 0.0203 | 0.0529 | 1.08×10^-3^ | 0.0515 | non-essential  (two genes) |
| Synpcc7942_0639 (*eno*) | enolase | 0.0154 | 0.0587 | 9.01×10^-4^ | 0.140 | essential |
| Synpcc7942_1760 (*ald*) | alanine dehydrogenase | 0.0220 | 0.0351 | 7.73×10^-4^ | 0.0680 | non-essential |
| Synpcc7942_2258 (*avtA*) | valine-pyruvate transaminase | 0.0231 | 0.0323 | 7.45×10^-4^ | 0.0648 | non-essential |
| cytochrome c oxidase genes  (*mcrA*, *ctaC*, *ctaD*, *ctaE*) | cytochrome c oxidase | 0.0237 | 0.0291 | 6.90×10^-4^ | 0.0527 | non-essential  (four genes) |
| Synpcc7942_0098 (*pyk2*) | pyruvate kinase | 0.0213 | 0.0319 | 6.78×10^-4^ | 0.0875 | essential |
| Synpcc7942_2503 (*por*) | protochlorophyllide oxidoreductase | 0.0250 | 0.0237 | 5.93×10^-4^ | 0.0515 | essential |
| Synpcc7942_1371 (*corA*) | magnesium and cobalt transport protein CorA | 0.0250 | 0.0237 | 5.93×10^-4^ | 0.0515 | non-essential |

Table S4. The essentiality of genes involved in NDH-1 complexes.

| **Gene ID** | **Gene name** | **Essentiality**  **(Rubin *et al*., 2015)** |
| --- | --- | --- |
| Synpcc7942_1343 | *ndhA* | essential |
| Synpcc7942_1415 | *ndhB* | essential |
| Synpcc7942_1180 | *ndhC* | essential |
| Synpcc7942_1976 | *ndhD1* | non-essential |
| Synpcc7942_1439 | *ndhD2* | beneficial |
| Synpcc7942_2092 | *ndhD3* | beneficial |
| Synpcc7942_0609 | *ndhD4* | non-essential |
| Synpcc7942_1473 | *ndhD5* | beneficial |
| Synpcc7942_1346 | *ndhE* | essential |
| Synpcc7942_1977 | *ndhF1* | beneficial |
| Synpcc7942_2091 | *ndhF3* | beneficial |
| Synpcc7942_0309 | *ndhF4* | non-essential |
| Synpcc7942_1345 | *ndhG* | essential |
| Synpcc7942_1743 | *ndhH* | essential |
| Synpcc7942_1344 | *ndhI* | beneficial |
| Synpcc7942_1182 | *ndhJ* | essential |
| Synpcc7942_1181 | *ndhK* | essential |
| Synpcc7942_1982 | *ndhM* | essential |
| Synpcc7942_2234 | - | essential |
| Synpcc7942_0278 | *hoxE* | non-essential |
| Synpcc7942_2557 | *hoxU* | non-essential |
| Synpcc7942_2257 | - | non-essential |

**References**

**Hirokawa, Y., Maki, Y., Hanai, T.,** 2016. Cyanobacterial production of 1,3-propanediol directly from carbon dioxide using a synthetic metabolic pathway. Metab. Eng. 34, 97–103.

**Hirokawa, Y., Maki, Y., Hanai, T.,** 2017. Improvement of 1,3-propanediol production using an engineered cyanobacterium, Synechococcus elongatus by optimization of the gene expression level of a synthetic metabolic pathway and production conditions. Metab. Eng. 39, 192-199.

**Rubin, B.E., Wetmore, K.M., Price, M.N., Diamond, S., Shultzaberger, R.K., Lowe, L.C., Curtin, G., Arkin, A.P., Deutschbauer, A., Golden, S.S.,** 2015. The essential gene set of a photosynthetic organism. Proc. Natl. Acad. Sci. U.S.A. 27, 6634-6643.
