# Supplementary material for: Metabolic engineering of Synechococcus elongatus PCC 7942 for improvement of 1,3-propanediol and glycerol production based on in silico simulation of metabolic flux distribution
Source: Microb Cell Fact. 2017 Nov 25;16:212. doi: 10.1186/s12934-017-0824-4 (PMC5702090; doi:10.1186/s12934-017-0824-4)
Supplement: Supplementary file 2 — Additional file 2. Additional figures. [file 12934_2017_824_MOESM2_ESM.ppt]

## Slide 1
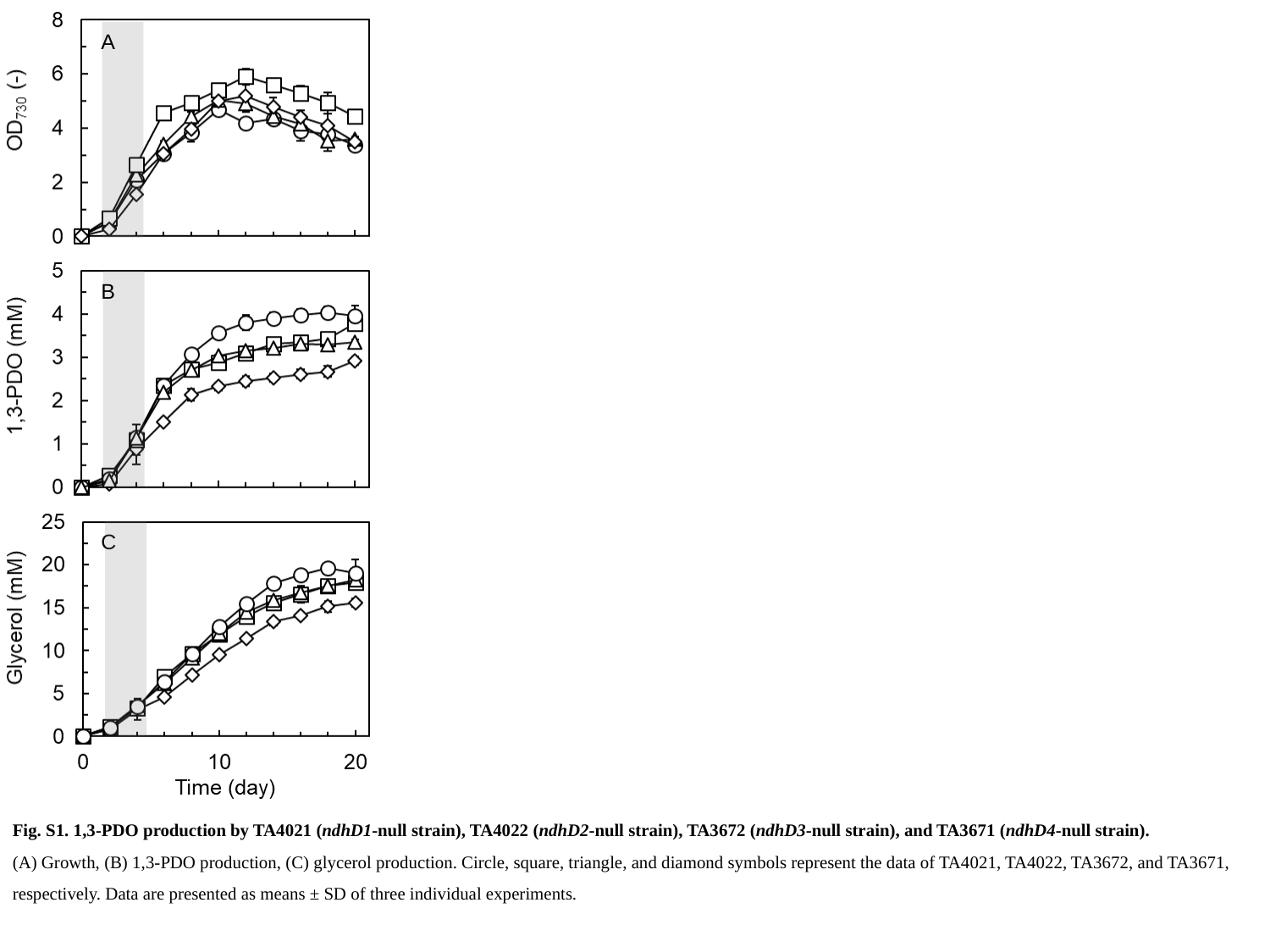

A
 B
 C
Fig. S1. 1,3-PDO production by TA4021 (ndhD1-null strain), TA4022 (ndhD2-null strain), TA3672 (ndhD3-null strain), and TA3671 (ndhD4-null strain).
(A) Growth, (B) 1,3-PDO production, (C) glycerol production. Circle, square, triangle, and diamond symbols represent the data of TA4021, TA4022, TA3672, and TA3671, respectively. Data are presented as means ± SD of three individual experiments.

## Slide 2
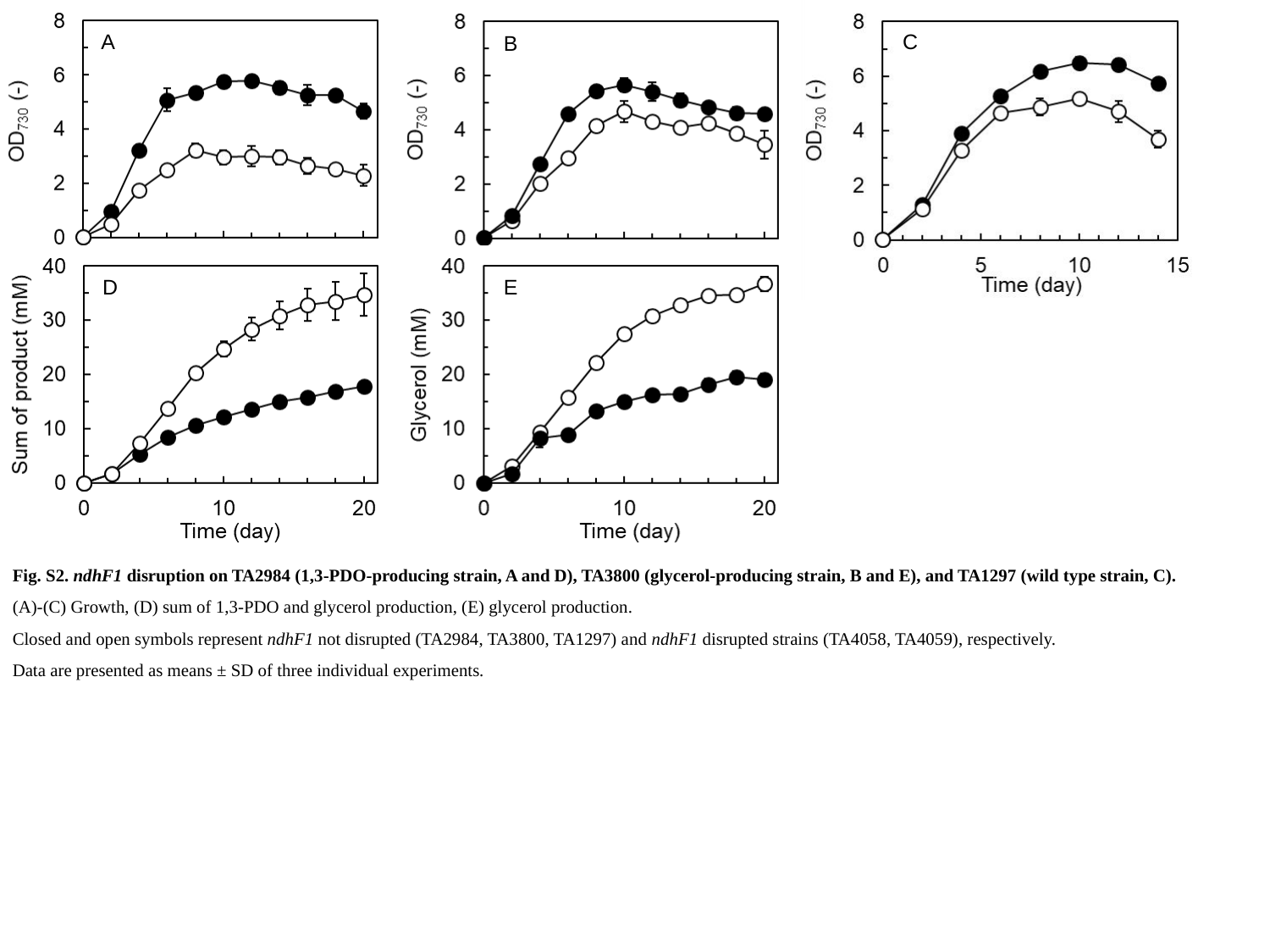

A
 C
 B
 D
 E
Fig. S2. ndhF1 disruption on TA2984 (1,3-PDO-producing strain, A and D), TA3800 (glycerol-producing strain, B and E), and TA1297 (wild type strain, C).
(A)-(C) Growth, (D) sum of 1,3-PDO and glycerol production, (E) glycerol production.
Closed and open symbols represent ndhF1 not disrupted (TA2984, TA3800, TA1297) and ndhF1 disrupted strains (TA4058, TA4059), respectively.
Data are presented as means ± SD of three individual experiments.

## Slide 3
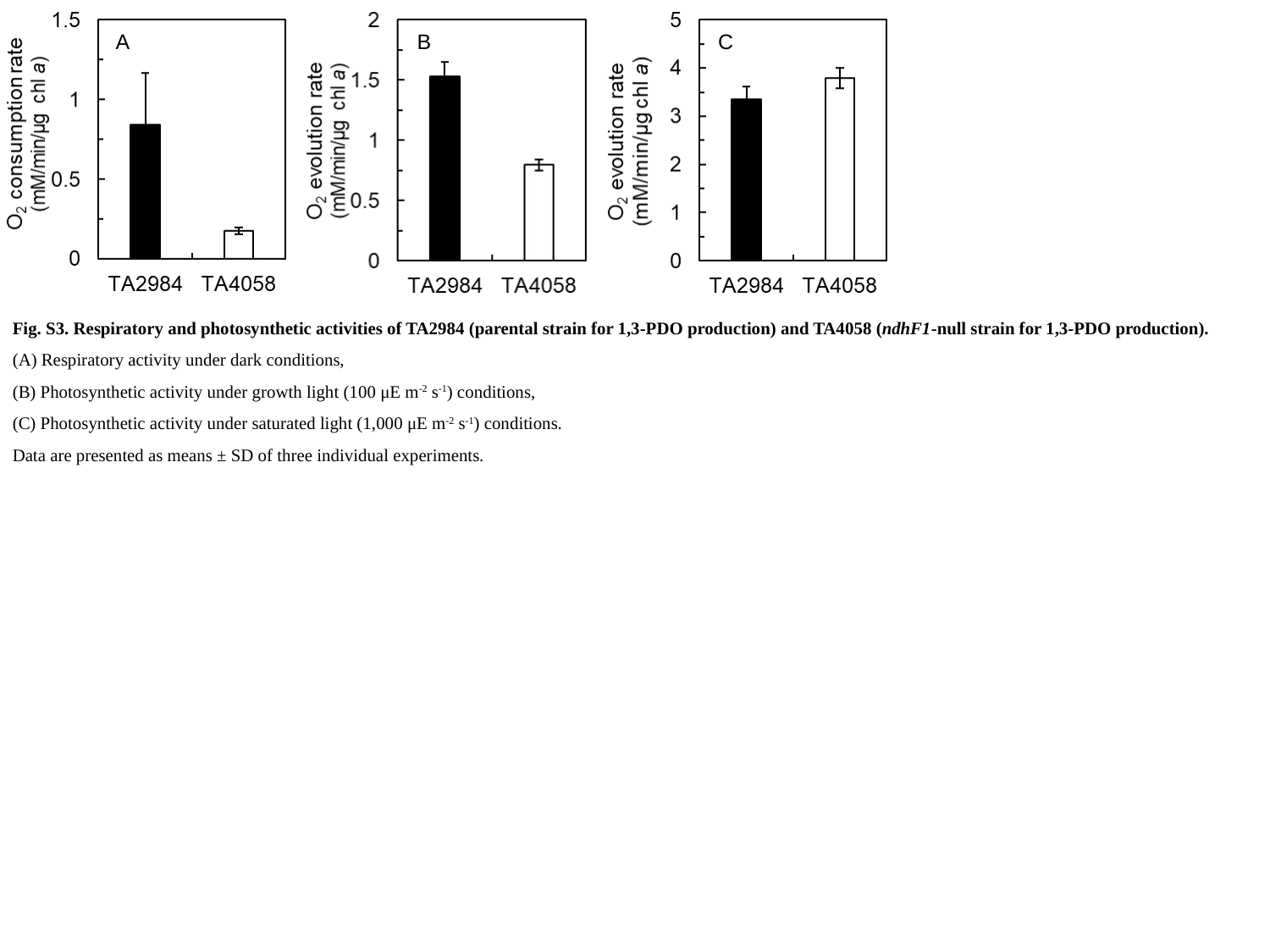

A
 B
 C
Fig. S3. Respiratory and photosynthetic activities of TA2984 (parental strain for 1,3-PDO production) and TA4058 (ndhF1-null strain for 1,3-PDO production).
(A) Respiratory activity under dark conditions,
(B) Photosynthetic activity under growth light (100 μE m-2 s-1) conditions,
(C) Photosynthetic activity under saturated light (1,000 μE m-2 s-1) conditions.
Data are presented as means ± SD of three individual experiments.

## Slide 4
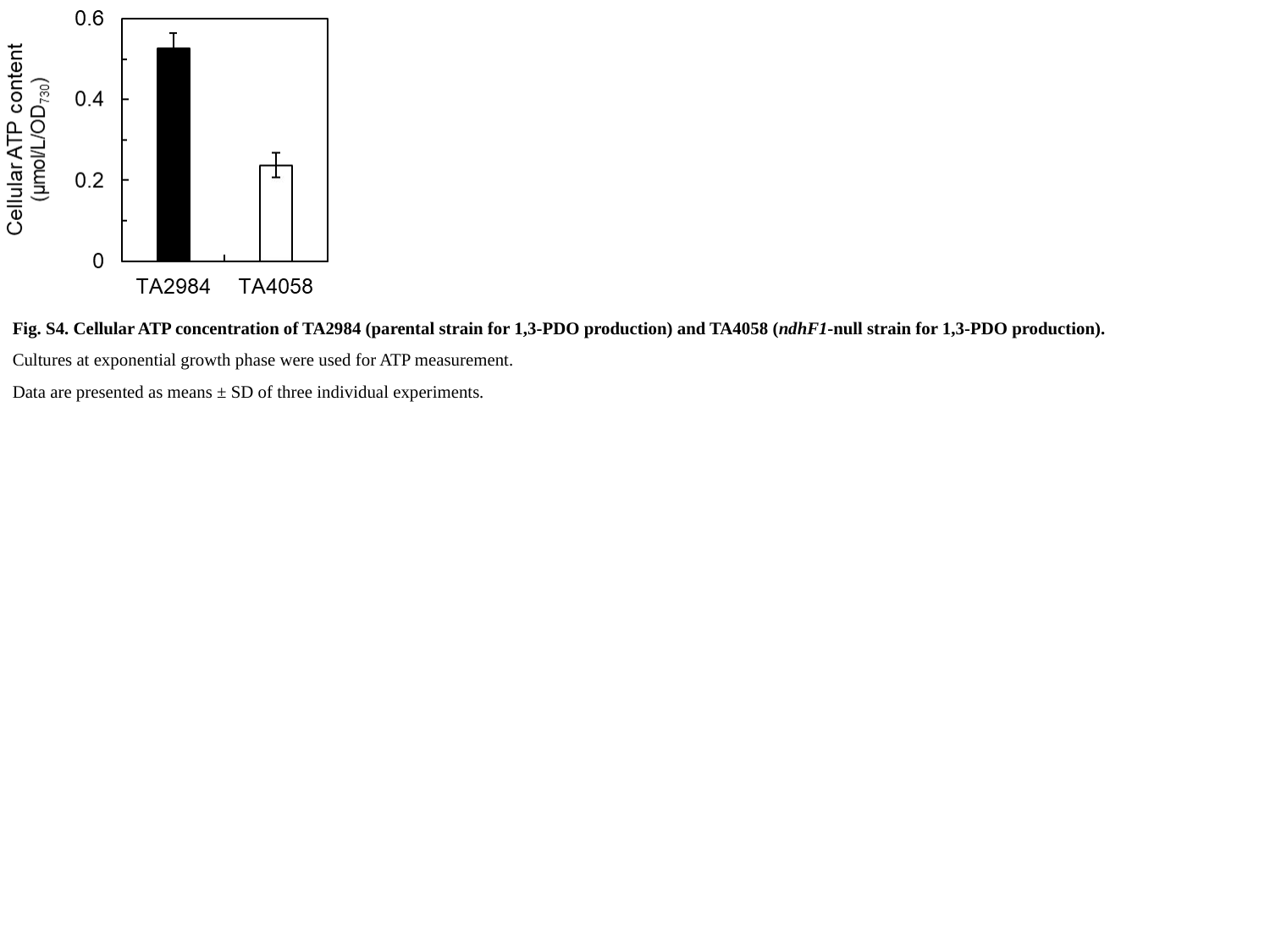

Fig. S4. Cellular ATP concentration of TA2984 (parental strain for 1,3-PDO production) and TA4058 (ndhF1-null strain for 1,3-PDO production).
Cultures at exponential growth phase were used for ATP measurement.
Data are presented as means ± SD of three individual experiments.

## Slide 5
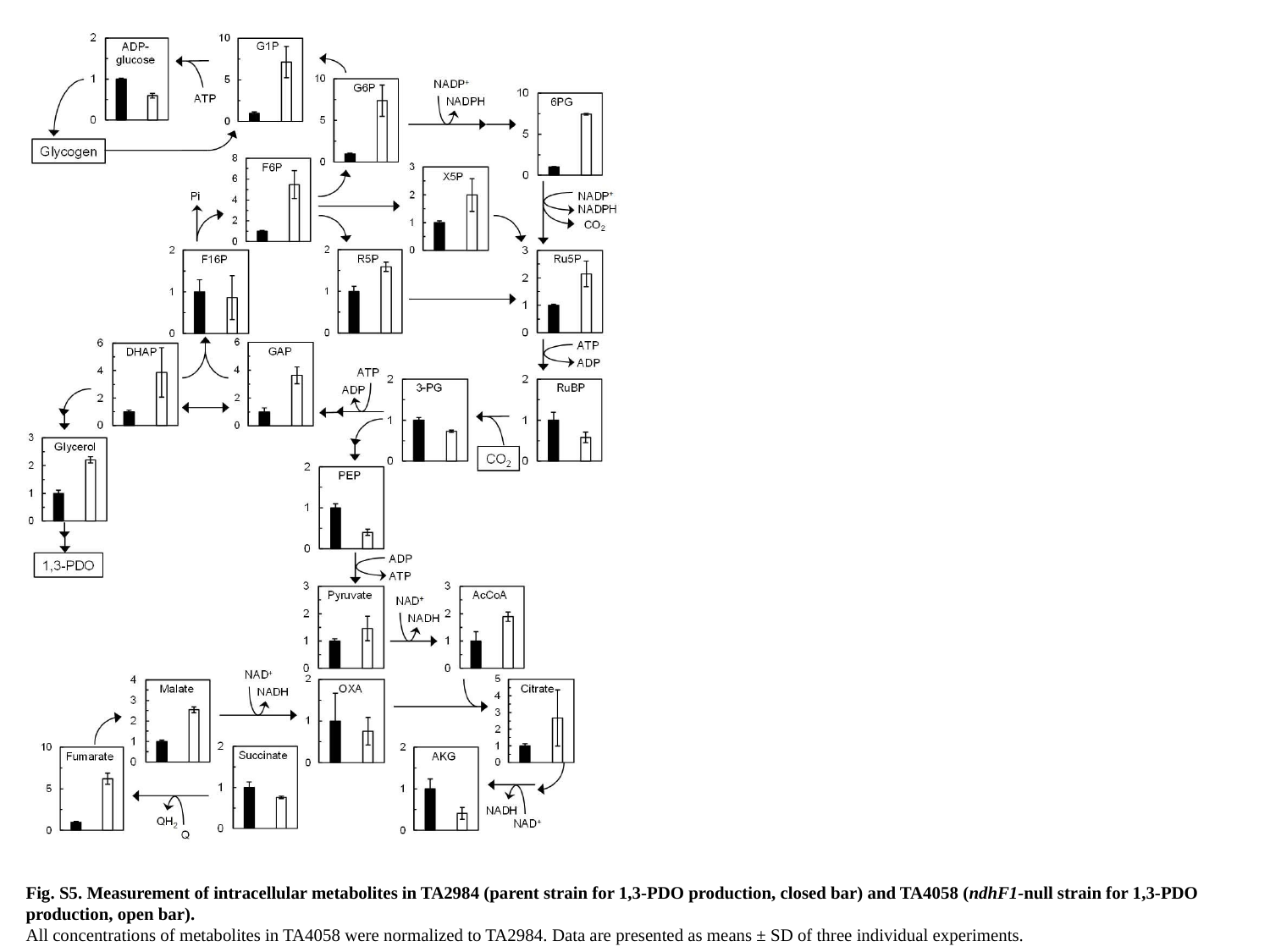

Fig. S5. Measurement of intracellular metabolites in TA2984 (parent strain for 1,3-PDO production, closed bar) and TA4058 (ndhF1-null strain for 1,3-PDO production, open bar).
All concentrations of metabolites in TA4058 were normalized to TA2984. Data are presented as means ± SD of three individual experiments.

## Slide 6
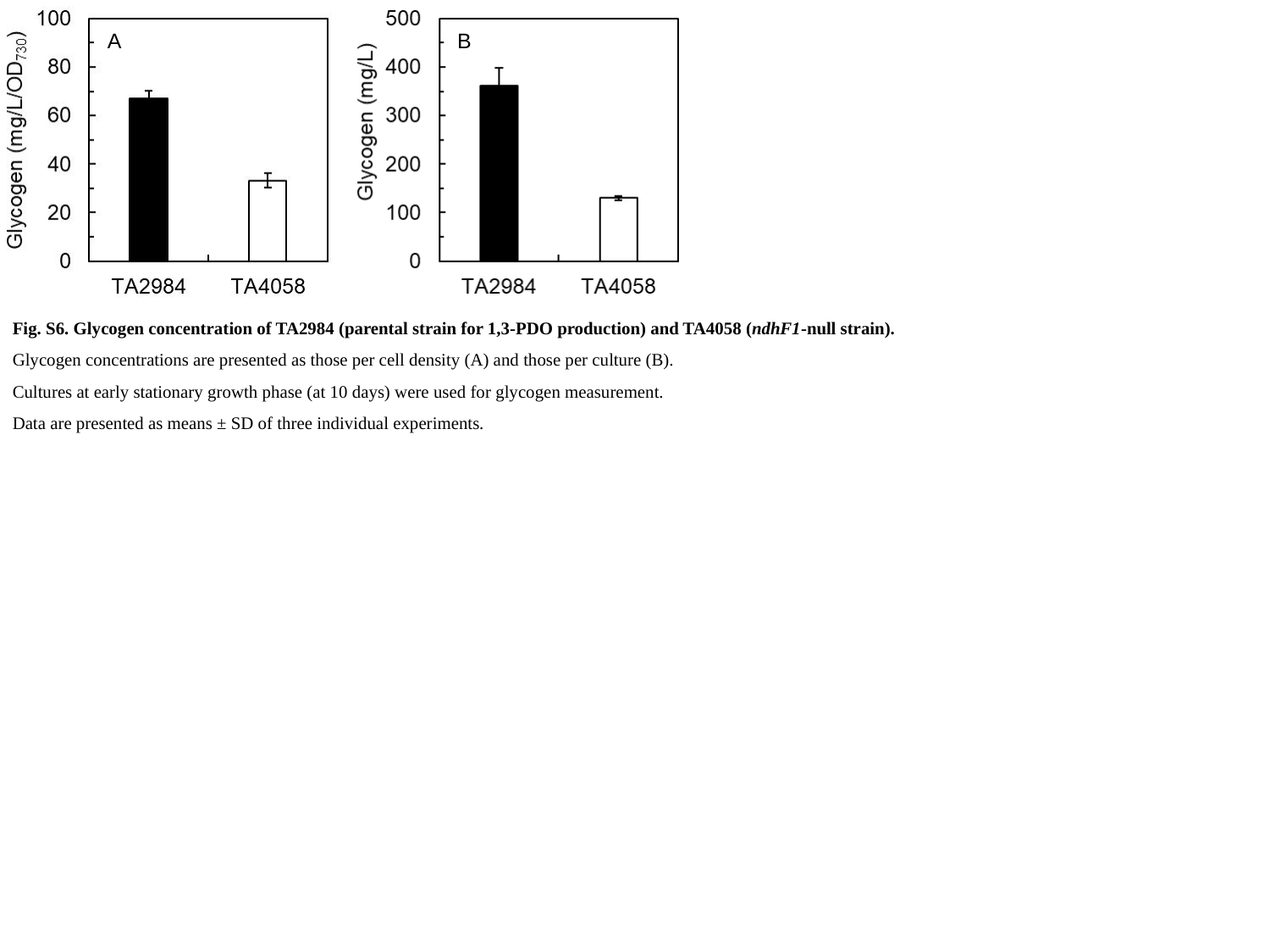

A
 B
Fig. S6. Glycogen concentration of TA2984 (parental strain for 1,3-PDO production) and TA4058 (ndhF1-null strain).
Glycogen concentrations are presented as those per cell density (A) and those per culture (B).
Cultures at early stationary growth phase (at 10 days) were used for glycogen measurement.
Data are presented as means ± SD of three individual experiments.
